# Environmental and Host Effects on Skin Bacterial Community Composition in Panamanian Frogs

**DOI:** 10.3389/fmicb.2018.00298

**Published:** 2018-02-22

**Authors:** Brandon J. Varela, David Lesbarrères, Roberto Ibáñez, David M. Green

**Affiliations:** ^1^Department of Biology, McGill University, Montreal, QC, Canada; ^2^Redpath Museum, McGill University, Montreal, QC, Canada; ^3^Smithsonian Tropical Research Institute, Panama City, Panama; ^4^Department of Biology, Laurentian University, Sudbury, ON, Canada; ^5^Departamento de Zoología, Universidad de Panamá, Panama City, Panama

**Keywords:** frog skin microbiota, metabarcoding, abiotic factors, *Dendrobates auratus*, *Silverstoneia flotator*, *Allobates talamancae*, chytrid, *Batrachochytrium dendrobatidis*

## Abstract

Research on the amphibian skin microbiota has focused on identifying bacterial taxa that deter a pathogenic chytrid fungus, and on describing patterns of microbiota variation. However, it remains unclear how environmental variation affects amphibian skin bacterial communities, and whether the overall functional diversity of the amphibian skin microbiota is associated to such variation. We sampled skin microbial communities from one dendrobatoid frog species across an environmental gradient along the Panama Canal, and from three dendrobatoid frog species before and after the onset of the wet season in one site. We found frog skin microbial alpha diversity to be highest in frogs from sites with low soil pH, but no clear effect of the onset of the wet season. However, we found frog skin microbial community structure to be affected by soil pH and the onset of the wet season, which also resulted in a decrease in between-sample variation. Across the sampled frog species, bacterial functional groups changed with the onset of the wet season, with certain bacterial functional groups entirely disappearing and others differing in their relative abundances. In particular, we found the proportion of Bd-inhibitory bacteria to correlate with mean soil pH, and to increase in two of the frog species with the onset of the wet season. Taken together, our results suggest that structure and predicted function of amphibian bacterial skin communities may be influenced by environmental variables such as pH and precipitation, site effects, and host effects.

## Introduction

The interactions between vertebrate hosts and their microbial communities have recently become a subject of accrued interest (Caporaso et al., 2010b, 2012; Escalona et al., 2016; Rebollar et al., 2016a), because microbiota may play an important role in animal ecology and evolution. For example, microbiota have been found to alter the behavior of their host (Bravo et al., 2011; Ezenwa et al., 2012; Marin et al., 2017), affect digestion (Schnorr et al., 2014) and development (McFall-Ngai et al., 2013), as well as contribute to immune system function (Kueneman et al., 2016a; Woodhams et al., 2016). However, how wildlife-associated microbiota vary according to abiotic factors remains poorly understood (Jiménez and Sommer, 2017; Medina et al., 2017; Pollock et al., 2017).

In amphibians, the skin microbiota are known to vary between host species (Kueneman et al., 2014; Belden et al., 2015; Pollock et al., 2017) and sampling sites (Kueneman et al., 2014; Krynak et al., 2016). It has also been suggested that ontogenetic and seasonal changes contribute to variation in the frog skin microbiota (Longo et al., 2015). Interestingly, microbes abundant on amphibians’ skin are usually rare or present in low abundances in the environment, suggesting that the amphibian skin represents a unique and selective environment (Loudon et al., 2014; Walke et al., 2014; Rebollar et al., 2016b). However, it is also clear that amphibians depend on microbial environmental reservoirs, such as forest soil, to maintain diverse skin microbiota (Fitzpatrick and Allison, 2014; Loudon et al., 2014; Kueneman et al., 2016a).

Both moisture (Hartmann et al., 2014) and pH (Fierer and Jackson, 2006; Lauber et al., 2009; Zhalnina et al., 2015; Schappe et al., 2017) drive patterns of variation in soil microbial communities, but whether these abiotic factors indirectly shape the amphibian skin microbial communities remains unclear. Alternatively, soil pH and moisture could potentially have an effect on microbe–microbe and microbe–host interactions, for example, by affecting amphibian antimicrobial peptide secretions (Rinaldi, 2002; Tennessen et al., 2009). Even though many frog skin microbiota studies were focused on identifying sources of variation, whether changes in skin microbial communities correspond with changes in overall microbial functional groups remains poorly understood.

In amphibians, the skin microbiota appear to constitute a critical component of the immune system (Walke et al., 2015; Woodhams et al., 2015; Kueneman et al., 2016b), whereby certain bacterial taxa are capable of producing anti-fungal metabolites (Brucker et al., 2008; Lauer et al., 2008; Harris et al., 2009b). Some of these anti-fungal metabolites deter the pathogenic chytrid fungus *Batrachochytrium dendrobatidis* (Bd), which has been associated with certain amphibian declines (Lips et al., 2006; Pounds et al., 2006). One of these agents, the betaproteobacterium *Janthinobacterium lividum* has been isolated from a number of amphibians (e.g., *Plethodon cinereus*, Brucker et al., 2008; Becker and Harris, 2010; *Gastrotheca excubitor*, Burkart et al., 2017), and can reduce Bd-related mortality (Harris et al., 2009a; Becker and Harris, 2010).

To date, only a few studies have empirically assessed variation in the overall microbial functional groups in amphibian hosts (Loudon et al., 2014; Davis et al., 2017). However, ecologically important the anti-Bd function of the skin microbes may be, this constitutes only one of the many functions performed by the amphibian skin microbiota. To have a better understanding of the functional implications of amphibian skin microbiota disruption, for example due to chytridiomycosis or environmental variation, it is necessary to better characterize the composition of these microbiota as a first step in assessing the broad spectrum of functionality that they may represent for the amphibian host. Accordingly, we sought to investigate the possible effects of abiotic environmental variables (i.e., soil pH and precipitation levels) across four sites in one frog species, as well as seasonal and host effects in three species of dendrobatoid frogs within one site in shaping the diversity and predicted function of the skin microbiota. Sites along the Panama Canal inhabited by these frogs differ in soil pH and annual precipitation, with well-defined wet and dry seasons, within a relative small area. Only about 50 km separate the Pacific from the Atlantic termini of the Panama Canal, along which is a steep climatic gradient in precipitation, with average annual precipitation ranging from ca. 1,500 to over 4,000 mm per year (Santiago and Mulkey, 2005; Engelbrecht et al., 2007).

If amphibians require microbial environmental reservoirs, such as forest soil, to maintain diverse skin bacterial communities (Loudon et al., 2014), then environmental variables that drive variation in free-living microbial communities like (1) soil pH, (2) precipitation levels, and (3) the onset of the wet season should correlate with frog skin microbial community structure. Frog skin bacterial diversity should be expected to be highest in frogs sampled from sites with neutral soil pH and high humidity, where soil bacterial diversity is highest (Fierer and Jackson, 2006; Lauber et al., 2009; Hartmann et al., 2014; Zhalnina et al., 2015), in comparison to either pH extreme and dryer sites. We also explored (4) host-specific differences on the skin microbiota. Finally, (5) if soil pH, precipitation levels, the onset of the wet season, and host-related skin microbial community shifts are ecologically relevant to these three dendrobatoid frog species, then we should observe a relationship between them and predicted microbial functional group variation, including the presence or absence of bacteria that may either inhibit or facilitate the growth of Bd.

## Materials and Methods

### Sampling Sites and Frog Species

**FIGURE 1:**
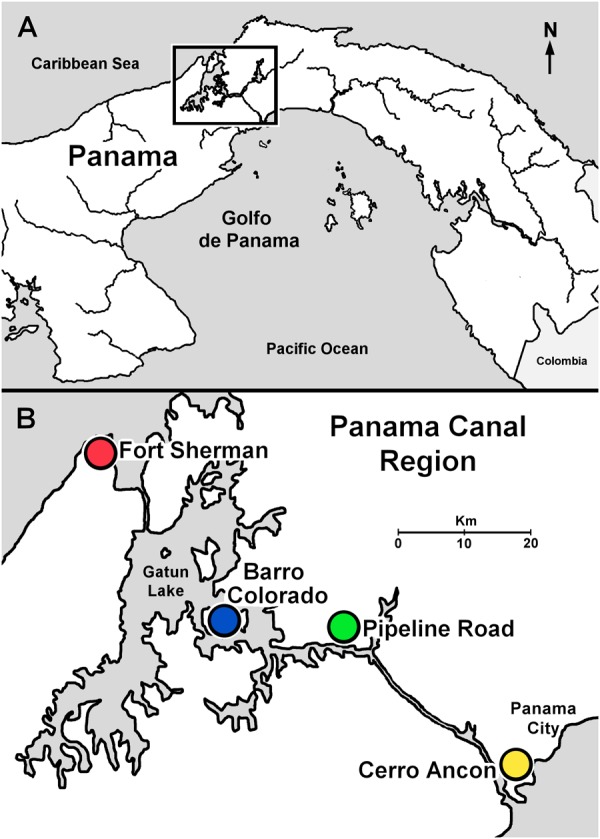
Sample sites along the Panama Canal in Central Panama. **(A)** Central Panama showing location of the Panama Canal region (inset). **(B)** Panama Canal region indicating sample localities.

In total, we sampled 70 dendrobatoid frogs (**Table 1**) from four sites along the Panama Canal in April 2016 (**Figure 1**). Cerro Ancon is a secondary growth urban forest, whereas the other three sites are characterized by old growth forests (Ibáñez et al., 2002). For each site, soil pH was recorded from soil samples (2–10 cm from surface) mixed in water (1:2, soil:water ratio; **Table 2**). We obtained historical annual mean and daily precipitation records from the Panama Canal Authority weather station closest to each site (20–136 years of records), and we used the daily values to calculate accumulated precipitation 5, 10, and 15 days before sampling. We used these accumulated precipitation values to confirm the beginning of the wet season. We believe that these time intervals could explain microbiota variation, as previous studies have recorded changes in amphibian skin microbiota in as little as 7 days in captivity (Loudon et al., 2014) and 3 days in field experiments (Longo and Zamudio, 2016).

**Table 1:** Host species habitat and sample sizes of the three dendrobatoid frogs by life stage.

Host species (sample size)	Host’s habitat	Site-specific sample sizes (before, after onset of wet season)			
		Fort Sherman	Cerro Ancon	Pipeline Road	Barro Colorado Island
*Dendrobates auratus* (58)	Leaf litter	8A, 1J	12A, 7J	9A, 2J (7A 1J, 2A 1J)	15A, 4J
*Silverstoneia flotator* (6)	Stream-associated			6A (2, 4)	
*Allobates talamancae* (6)	Stream-associated			6A (2, 4)	

*Life stage: A, adult frogs or J, juvenile frogs.*

**Table 2:** Abiotic factors for the sites sampled along the Panama Canal: soil pH and precipitation levels.

Site (sampling date)	Mean soil pH ± SE (*n*)	Prec. 5/10/15	Mean annual precipitation
Fort Sherman (4 April 2016)	4.7 ± 0.3 (26)	0/0/2	255.5
Cerro Ancon (7 April 2016)	5.4 (1)	0/0/0	153.3
Pipeline road^∗^ (9 and 23 April 2016)	5.6 ± 0.3 (17)	0/0/07/7/8	178.9
Barro Colorado Island (11 and 12 April 2016)	6.2 ± 0.5 (10)	1/1/1	204.9

*^∗^We resampled Pipeline Road as soon as the rainy season started; thus, we provide two sets of cumulative precipitation values.*

To test the effects of soil pH and precipitation levels on skin microbiota, we focused our sampling on juvenile and adult *Dendrobates auratus* (*N* = 9–19 per site, four sites), a frog living in the leaf litter where soil pH is likely affecting its microbiota (dataset “Dend.aura”). We included *D. auratus* life stage as a fixed factor in our models to account for previously reported ontogenetic differences (Longo et al., 2015). To test seasonal effects on skin microbiota in one site (Pipeline Road), we sampled three dendrobatoid frog species before and after the onset of the wet season (*D. auratus N*_before_ = 8, *N*_after_ = 3; *Silverstoneia flotator N*_before_ = 2, *N*_after_ = 4; and *Allobates talamancae N_before_* = 2, *N*_after_ = 4, dataset “Pipeline”). To test differences between frog species, we used the “Pipeline” dataset and controlled for sampling time (i.e., “before” or “after” the onset of the wet season).

To collect our samples, we caught frogs with gloved hands and rinsed them with 50 ml of distilled autoclaved water to wash off any transient microbes (Loudon et al., 2014; Rebollar et al., 2016b). After rinsing, we swabbed the frogs with two sterile cotton tipped swabs 10 times on the ventral side, 10 times on each leg and each toe once. We used new gloves for each frog to prevent cross-contaminating our samples. We stored the swabs in sterile, autoclaved micro-centrifuge tubes on ice until they could be stored in a -20°C freezer prior to DNA extraction. We used one swab for DNA extraction to test Bd presence and to construct 16S rRNA libraries. We stored the second swab at -80°C as a reference.

Research permits allowing the collection of our samples were granted by the Panamanian authority Ministerio de Ambiente (Permit No. SE/A-38-16) and by the Authority of the Panama Canal (Permit No. 173). All procedures with animals were conducted under approved animal care protocols from the Smithsonian Tropical Research Institute’s Animal Care Committee (2016-0301-2019-2) and by the McGill University’s Animal Care Committee (2000-4569).

### DNA Extraction, Bd Analyses, and 16S rRNA Library Preparation

We treated swabs with a lysozyme buffer to break up Gram-positive bacteria’s cell walls, followed by a modified protocol of the QIAGEN DNeasy Blood & Tissue Kit.

To test for Bd presence, we performed the qPCR assay developed by Boyle et al. (2004) with the following modifications. We extracted DNA from swabs using the QIAGEN DNeasy Blood & Tissue Kit (Kosch and Summers, 2013; Bletz et al., 2015). We performed the qPCR reactions on a Roche LightCycler 96 System, and a volume of 20 μl per well (Kriger et al., 2006a). Instead of using Applied Biosystems 2× TaqMan Master Mix, we used Roche FastStart Essential DNA Probes Master and we added 0.25 μl of Roche LightCycler Uracil-DNA Glycosylase per reaction to eliminate potential PCR carryover contamination. We used Applied Biosystems TaqMan Exogenous Internal Positive Control Reagents VIC Probe (Hyatt et al., 2007), and we included five negative and two Bd-positive controls on every plate (Kriger et al., 2006a,b). We performed a duplicate analysis by initially running a 1:10 dilution of the DNA extract from each sample in singlicate, followed by a second run of the undiluted DNA extract in singlicate to avoid false negatives due to a potentially low and undetectable quantity of DNA in the diluted samples. Samples in which Bd was not detected in both runs of the duplicate analysis were considered negative. Despite the fact that the sites we sampled have previously been reported as Bd endemic (Woodhams et al., 2008; Rebollar et al., 2014), and Bd might still be present in other amphibian species, all our samples tested negative for Bd; therefore, we did not include Bd as a factor in our statistical analyses.

To construct our 16S rRNA library, we amplified each sample following Caporaso et al. (2012), using primers F515 and R806. We pretreated the PCR pools with Just-a-Plate^TM^ PCR 96 Purification and Normalization Kit (Charm Biotech, San Diego, CA, United States). We used equimolar parts of each sample to construct the library and cleaned it with Serapure beads to remove any primer dimers. DNA concentration and quality of the library were assessed with NanoDrop^TM^ ND-1000 (Thermo Fisher Scientific Inc., 2008, Wilmington, DE, United States), Invitrogen Qubit^®^ Fluorometer (Thermo Fisher Scientific Inc., 2007, Wilmington, DE, United States), and Agilent 2100 Bioanalyzer (Agilent Technologies, Inc., 2000–2005, Santa Clara, CA, United States). The library was sequenced at the NAOS laboratories of the Smithsonian Tropical Research Institute in Panama using an Illumina MiSeq v3 600 cycles cartridge.

### Sequence Reads Processing

We processed all samples in the same manner before separating them into the two data sets. Using Quantitative Insights into Microbial Ecology (QIIME, Caporaso et al., 2010b), we filtered sequences to retain high-quality reads. We clustered sequences according to 97% sequence similarity threshold using the UCLUST method (Edgar, 2010). The resulting operational taxonomic units (OTUs) were classified using the Greengenes database when possible (May 2013 release; McDonald et al., 2012); otherwise they were clustered *de novo* using a 97% sequence similarity threshold. We assigned taxonomy with the Greengenes database and the RDP classifier (Wang et al., 2007). We used PyNAST (Caporaso et al., 2010a) to align representative sequences to the Greengenes database and constructed a phylogenetic tree with FastTree2 (Price et al., 2010). Lastly, we filtered the resulting OTU table to only include clusters ≥0.001% of the total reads (Bokulich et al., 2013). To standardize sequencing effort, we rarefied the samples to a depth of 6,813 sequences. This initial dataset included 27,120 bacterial OTUs from 70 samples. As demonstrated by plateauing rarefaction curves, the samples had appropriate sequencing coverage, which indicates that our sampling captured most of the bacterial alpha diversity (Supplementary Figure S1). For downstream analyses, we divided the initial dataset into a subset of 22,846 OTUs from 58 samples of *D. auratus* from the four sites (“Dend.aura”), and a subset of 12,354 OTUs from 23 samples from all three species from Pipeline Road (“Pipeline”). Sequences for each sample were deposited in the NCBI SRA under BioProject ID PRJNA433445.

### Comparing Alpha and Beta Diversity

Unless otherwise stated, all statistical and graphical analyses were performed using R 3.3.2 (R Core Team, 2017, Vienna, Austria) and the *ggplot2* package (Wickham, 2009). Full test statistics can be found in Supplementary Table S1.

To estimate alpha diversity of skin microbiota per frog species and site, we calculated Shannon’s diversity index, which measures the number of species and their evenness within a sample. Using the *lme4* package (Bates et al., 2015) and the “Dend.aura” dataset, we tested for an effect of mean soil pH and precipitation levels on bacterial alpha diversity, including site as a random factor and life stage as a fixed factor. We tested for an effect of the onset of the wet season on alpha diversity using unpaired *t*-tests (two-tailed) for each of the three host species from Pipeline Road (“Pipeline”) sampled before and after the onset of the wet season. We also tested for host effects on bacterial alpha diversity among the Pipeline Road sample using linear mixed effects model with sampling time as a random factor (i.e., “before” or “after” the onset of the wet season).

We used the data derived from *D. auratus* (“Dend.aura”) samples to test the effect of mean soil pH and precipitation levels on bacterial beta diversity, and data from Pipeline Road (“Pipeline”) samples of all three host species to test the effect of the onset of the wet season and host species effects on bacterial beta diversity. We calculated the UniFrac Weighted and UnWeighted (Lozupone and Knight, 2005) and Bray–Curtis distances matrices, and obtained NMDS ordinations. To determine differences between groups, we used the *adonis* function within the *vegan* package (Oksanen et al., 2016) and 999 permutations. We controlled for site effects by including site as *strata*, which does not correspond directly to a random factor, but in this case *strata* controlled for repeated measures within sites.

To determine which OTUs were driving the reported beta diversity differences, we performed linear discriminant analyses of effect sizes using LEfSe and normalizing the sum of values to 1M as recommended by Segata et al. (2011). We used 0.05 thresholds for both the Kruskal–Wallis (among class differences) and Wilcoxon (between classes differences) tests, and we ran a linear discriminant analysis (LDA) considering LDA scores >3 as ecologically relevant. LDA scores are an estimate of the effect size of features that are differentially abundant among the categories tested (Segata et al., 2011). For bacterial OTUs beta diversity and functional group richness analyses, we tested whether groups differed in their dispersion patterns by running the *betadisper* function from the *vegan* package (Oksanen et al., 2016), which is equivalent to a Levene’s test comparing homogeneity of variances between groups. We only reported significant *betadisper* results.

### Comparing Bacterial Diversity and Predicted Functional Group Variation

To assess whether the alpha and beta diversity differences associated with sites that differed in soil pH and precipitation levels, timing in relation to the onset of the wet season, and host species corresponded with changes in predicted microbial functional groups, we compared our OTU table against the Functional Annotation of Prokaryotic Taxa (FAPROTAX) database (Louca et al., 2016). FAPROTAX extrapolates functions of cultured prokaryotes (identified at the genus or species level) to the rest of the prokaryotic genus to estimate putative function. There are two main limitations of applying this approach to our data: (1) the FAPROTAX database was constructed mainly to analyze biogeochemistry of water bodies and (2) the FAPROTAX database is non-exhaustive; thus, only a small percentage of our OTUs may be assigned to at least one functional group. Even considering these caveats, we believe that predicting microbial functional groups using FAPROTAX is an appropriate alternative, especially when a metatranscriptomics approach is not plausible. Additionally, because FAPROTAX only considers cultured bacterial species with characterized functions, it may be superior to genomic prediction approaches like PICRUSt (Langille et al., 2013) and PAPRICA (Bowman and Ducklow, 2015).

### Estimating the Bd-Inhibitory and Bd-Enhancing Functions of the Skin Microbiota

Finally, to identify bacterial isolates that potentially inhibit or enhance Bd, we used the *pick_closed_reference_otus.py* script in QIIME (Caporaso et al., 2010b) and standard settings to compare our results to the database published by Woodhams et al. (2015). It should be noted that (a) the database only includes culturable bacteria and thus is not exhaustive, (b) the database is likely biased toward bacterial isolates that inhibit Bd, (c) some of the bacteria were isolated from non-Panamanian frogs (Woodhams et al., 2015), and (d) microbiota functions may be context dependent (Medina et al., 2017). To standardize the resulting OTU table, we calculated proportion of Bd-inhibitory and Bd-enhancing bacterial sequences per sampled frog. To do this, we considered each frog and computed the sum of the relative abundances of all Bd-inhibitory and Bd-enhancing bacteria separately. We compared these proportions between host species, sites, and in relation to the onset of the wet season using generalized linear models with the function *glm* (R Core Team, 2017). Because we were comparing proportions bound between 0 and 1 and to account for overdispersion, we used quasibinomial distributions in the case of Bd-inhibitory bacteria. Due to their low proportions, we used quasipoisson distributions to analyze Bd-enhancing bacteria.

## Results

### Among-Site Skin Microbiota Variation in *D. auratus*

**FIGURE 2:**
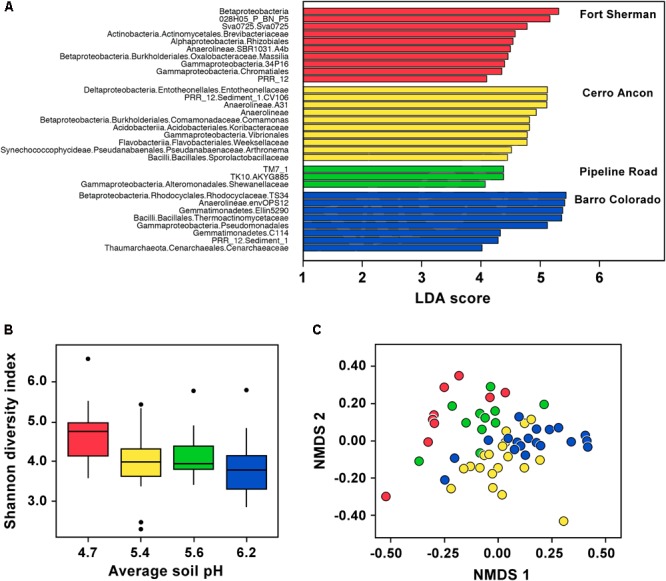
Skin microbiota in *Dendrobates auratus* in relation to sample site and soil pH. **(A)** Bacterial operational taxonomic units (OTUs) that best define bacterial community structure in *D. auratus* across sites. Bars represent most significant linear discriminant analysis (LDA) scores computed using LEfSe, i.e., LDA > 3. **(B)** Box-and-whisker plot comparison of skin bacterial Shannon alpha diversity in *D. auratus* across sites in relation to average soil pH. Box indicates median, first, and third quartiles; points extending past 1.5 times the inter-quartile length were considered outliers and depicted as dots. **(C)** Non-metric multi-dimensional scaling (NMDS) plot of Bray–Curtis distances in skin bacterial beta diversity between individuals of *D. auratus* across sites. In all charts, sites are color-coded as follows: Fort Sherman is red, Cerro Ancon is yellow, Pipeline Road in green, and Barro Colorado is dark blue.

In *D. auratus*, Shannon diversity correlated negatively with mean soil pH and was highest in frogs sampled from the site with lowest soil pH (**Figure 2B**, *P* < 0.05). However, precipitation levels did not have an effect on Shannon diversity (*P* > 0.05; nor on observed OTUs or Phylogenetic Diversity, Supplementary Table S1). In terms of bacterial community structure, we found the sites with the lowest mean soil pH (Fort Sherman) and highest mean soil pH (Barro Colorado Island) to plot on opposite sides of the ordination, with both intermediate mean soil pH sites (Cerro Ancon and Pipeline Road) in the middle. The pattern is less clear with regards to precipitation levels. The two sites with highest precipitation (Fort Sherman and Barro Colorado Island) plotted on opposite sides of the ordination, whereas the two intermediate precipitation level sites (Cerro Ancon and Pipeline Road) plotted in the middle of the ordination. Nevertheless, we found soil pH and precipitation levels as statistically significant factors explaining variation in bacterial community structure (**Figure 2C**, PERMANOVA UniFrac Weighted, Bray–Curtis, and UniFrac UnWeighted, *P* < 0.05). Out of 2,084 OTUs, we found 33 most significant OTUs best explaining site differences in *D. auratus* skin bacterial beta diversity (LDA > 3, **Figure 2A**). In particular, we found clear overrepresentation of *Pseudomonadaceae, Oxalobacteraceae*, and *Xanthomonadaceae* in Fort Sherman; *Sphingomonadaceae* and *Weeksellaceae* in Cerro Ancon; *Brevibacteriaceae* in Pipeline Road; and *Moraxellaceae* and *Sphingobacteriaceae* in Barro Colorado.

At higher bacterial taxonomic levels, though, the bacterial skin communities among *D. auratus* populations appeared to be similar. To illustrate, OTUs belonging to the Gammaproteobacteria were abundant in all sites, whereas Betaproteobacteria were abundant in all sites except in Pipeline Road. Anaerolineae bacteria were abundant in Fort Sherman and Barro Colorado Island, the two sites with the lowest and highest mean soil pH, respectively, among the four sites. Bacilli were abundant in Cerro Ancon and in Barro Colorado Island, which are the sites with the most human traffic (i.e., tourists and residents in Cerro Ancon, and researchers and visitors in Barro Colorado Island). The rest of the overrepresentations at higher taxonomic levels were site-specific. In Fort Sherman, Actinobacteria and Alphaproteobacteria were abundant; whereas Deltaproteobacteria, Acidobacteria, Flavobacteriia, and Pseudanabaenales were abundant in Cerro Ancon. At this taxonomic resolution, we did not detect any site-specific overrepresentations in Pipeline Road. Gemmatimonadetes and Thaumarchaeota were abundant in Barro Colorado Island.

### Within-Site Skin Microbiota Variation in Relation to the Wet Season and Host Species

**FIGURE 3:**
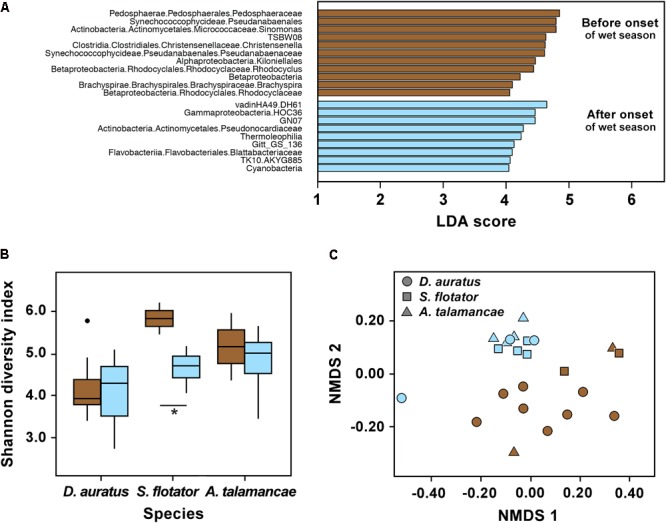
Skin microbiota in three dendrobatoid frog species from Pipeline Road in relation to the onset of the wet season. **(A)** Bacterial OTUs that best define bacterial community structure among species before and after the onset of the wet season. Bars represent most significant linear discriminant analysis (LDA) scores computed using LEfSe, i.e., LDA > 3. **(B)** Box-and-whisker plot comparison of skin bacterial Shannon alpha diversity in dendrobatoid frog species before and after the onset of the wet season. Box indicates median, first, and third quartiles; points extending past 1.5 times the inter-quartile length were considered outliers and depicted as dots. ^∗^ indicates a significant difference in alpha diversity before and after the onset of the wet season. **(C)** NMDS plot of Bray–Curtis distances in skin bacterial beta diversity between individual frogs of three dendrobatoid species at Pipeline Road. In all charts, brown indicates before the onset of the wet season, whereas light blue indicates after the onset of the set season.

We found no effect of the onset of the wet season on skin bacterial alpha diversity of Pipeline Road *D. auratus* or *A. talamancae* (both *P >* 0.05), but there was a barely significant decline in alpha diversity of *S. flotator* (**Figure 3B**; *t* = 2.77, *df* = 4, *P* = 0.05). Within Pipeline Road, we found frog skin bacterial beta diversity to be assorted into distinct clusters in relation to the onset of the wet season (**Figure 3C**; PERMANOVA UniFrac Weighted and UniFrac UnWeighted *P* < 0.001; Bray–Curtis *P* < 0.01). After the onset of the wet season, the frog skin microbiota became more homogeneous, which was illustrated by the wet season samples forming a tighter cluster than the dry season samples (**Figure 3C**; *betadisper* ANOVA on UniFrac UnWeighted distances *F*_(1,21)_ = 6.791, *P* < 0.05). Out of 1,679 OTUs, we found 20 most significant OTUs explaining the differences in relation to the onset of the wet season (LDA > 3, **Figure 3A**). Actinobacteria OTUs were abundant both before and after the onset of the wet season. However, the abundant Actinobacteria before the onset of the wet season was a Micrococcaceae, whereas after the onset of the wet season the abundant OTU belonged to the Pseudonocardiaceae. The frogs sampled before the onset of the wet season were characterized by an abundance of OTUs belonging to Pedosphaerae, Synechococcophycidae, Clostridia, Alphaproteobacteria, Betaproteobacteria, and Brachyspirae. On the other hand, Gammaproteobacteria, Thermophilia, Flavobacteriia, and Cyanobacteria were abundant after the onset of the wet season. Overall, some bacterial classes appeared to be more abundant before the onset of the wet season (e.g., Actinobacteria and Bacilli), and decreased after the onset of the wet season when other classes became relatively more abundant (e.g., Flavobacteria and Sphingobacteria, Supplementary Figure S2).

We found Shannon diversity to differ between frog species in Pipeline Road (*P* < 0.05). However, these three species did not differ in observed OTUs or Phylogenetic Diversity (Supplementary Table S1). Out of the three frog species sampled, *D. auratus* had the lowest alpha diversity indexes, followed by *A. talamancae* and then *S. flotator*. Across frog species, microbial communities were dominated by Gammaproteobacteria, Alphaproteobacteria, and Betaproteobacteria (Supplementary Figure S2). The two stream-associated frogs, *A. talamancae* and *S. flotator*, also appeared to have higher relative abundances of Sphingobacteria than *D. auratus*. Flavobacteria and Actinobacteria seemed to be more abundant in *D. auratus*. After controlling for sampling time effects, we found no host effect on beta diversity (PERMANOVA Bray–Curtis, UniFrac Weighted and UnWeighted *P* > 0.05).

### Skin Microbiota Predicted Function Variation

We could assign 489 out of 2,184 bacterial OTUs (22.39%) to at least one microbial functional group using the FAPROTAX database, and most functional groups were represented by more than one OTU (**Table 3**). *D. auratus* skin microbiota had different microbial functional groups at sites differing in soil pH and precipitation levels (PERMANOVA Bray–Curtis *P* < 0.001). These site-to-site differences were best explained by 17 functional groups (**Table 3**), including fermentation in Fort Sherman, aerobic chemoheterotrophy in Cerro Ancon, anoxygenic photoautotrophy in Pipeline Road, and human pathogens in Barro Colorado Island.

**Table 3:** Bacterial functional groups that best defined the site differences in the microbiota of *Dendrobates auratus* frogs between sampling sites, the number of bacterial operational taxonomic units (OTUs) identified for each functional group, and the relative abundance of bacteria in each functional group, presented in terms of linear discriminant analysis (LDA) scores computed using LEfSe.

Bacterial functional group	OTUs assigned	Relative abundance (LDA score)			
		Fort Sherman	Cerro Ancon	Pipeline Road	Barro Colorado Island
Chemoheterotrophy	294		4.500		
Aerobic chemoheterotrophy	199		4.569		
Phototrophy	90		3.954		
Photoautotrophy	84		4.219		
Fermentation	80	4.275			
Cyanobacteria	79		4.081		
Oxygenic photoautotrophy	79		4.081		
Intracellular parasites	39	3.793			
Animal parasites or symbionts	22				4.563
Chloroplasts	18	4.143			
Ureolysis	10			3.180	
Nitrogen fixation	8		3.158		
Human pathogens	5				4.567
Anoxygenic photoautotrophy sulfur oxidizing	5			3.819	
Anoxygenic photoautotrophy	5			3.819	
Aromatic compound degradation	3				4.555

**FIGURE 4:**
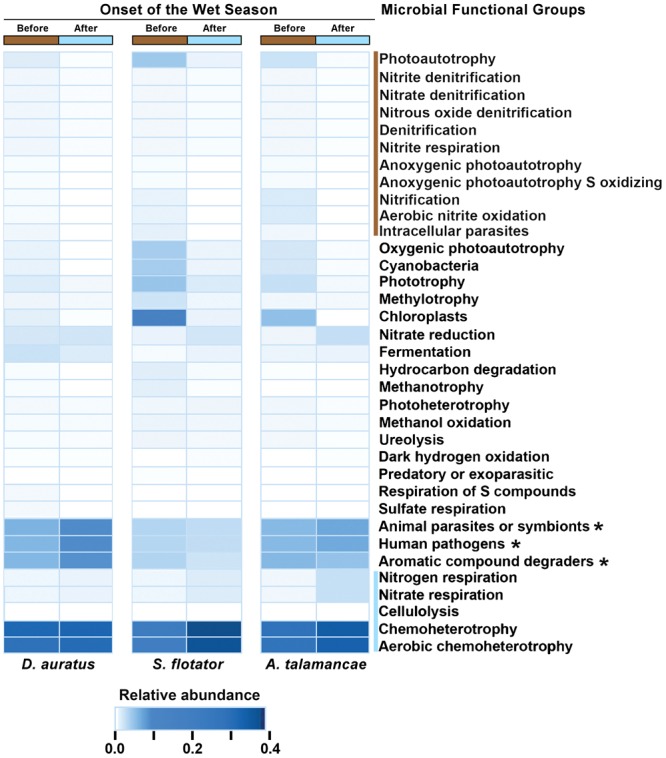
Mean relative abundances of skin microbial functional groups before and after the onset of the wet season in three species of dendrobatoid frogs, *Dendrobates auratus, Silverstoneia flotator*, and *Allobates talamancae*, from Pipeline Road, Panama. Relative abundances are depicted in terms of color intensity from white (=0) to darkest blue (=0.433). Microbial functional groups more abundant before the onset of the wet season are preceded by a brown bar, whereas groups more abundant after the onset of the wet season are preceded by a blue bar. Asterisks (^∗^) indicate microbial functional groups more abundant in *D. auratus*.

At Pipeline Road and after controlling for frog species, skin microbial functional groups were different before and after the onset of the wet season (PERMANOVA Bray–Curtis *P* < 0.01), with 16 functional groups associated with this difference (**Figure 4**). These differences were explained by certain functional groups changing in their relative abundance or entirely disappearing after the onset of the wet season. To elaborate, functional groups involving nitrite, nitrate, and photoautotrophy were more abundant before the onset of the wet season, whereas chemoheterotrophy, cellulolysis, and nitrogenous compounds respiration were more abundant after the onset of the wet season.

### Variation of Potential Bd-Interacting Microbiota

**FIGURE 5:**
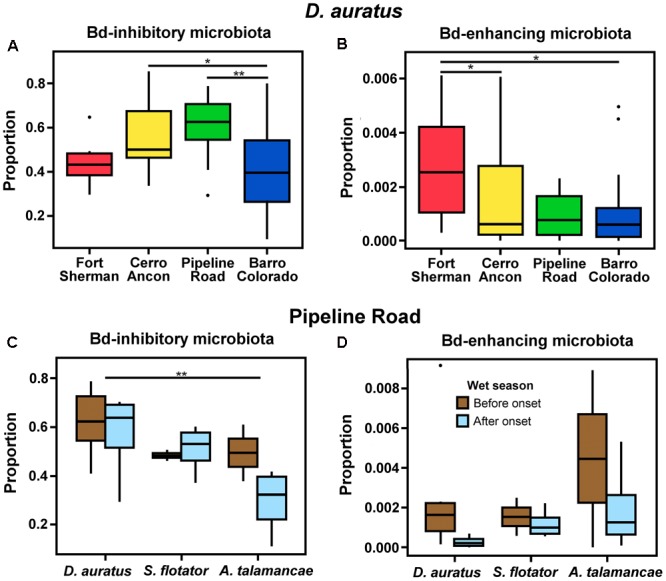
Proportions of *Batrachochytrium dendrobatidis* (Bd) inhibitory **(A)** and enhancing **(B)** microbiota in *D. auratus* across sites in central Panama, arranged in order of increasing average soil pH values **(A,B)**, and in relation to the onset of the wet season in three Panamanian dendrobatoid frog species at Pipeline Road **(C,D)**.

We could assign 185 out of 2,184 OTUs (8.47%) to one of the Bd-interacting categories using the Woodhams et al. (2015) database. The majority of these OTUs were isolated from Panamanian frogs (118/185 or 63.38%), and 14.05% (26/185) were isolated from the same species we report in this study (3 from *A. talamancae*, 6 from *D. auratus*, and 17 from *S. flotator*). *D. auratus* sampled from different sites differed in the proportion of Bd-inhibitory (**Figure 5A**, $\chi_{57,3}^{2}$ = 6.144, *P <* 0.010) and Bd-enhancing microbiota (**Figure 5B**, $\chi_{57,3}^{2}$ = 0.196, *P <* 0.05); yet, the two Bd-interacting microbial categories followed opposite trends. Bd-inhibitory bacterial OTUs were relatively more abundant in *D. auratus* from Pipeline Road, followed by Cerro Ancon, Fort Sherman, and Barro Colorado, whereas Bd-enhancing bacterial OTUs were relatively more abundant in Fort Sherman, followed by Cerro Ancon, Pipeline Road, and Barro Colorado Island.

In Pipeline Road, the proportion of Bd-inhibitory bacteria differed in relation to the onset of the wet season ($\chi_{22,1}^{2}$ = 2.420, *P <* 0.05) and between frog hosts (**Figure 5C**, $\chi_{20,2}^{2}$ = 2.420, *P* < 0.05). Even though Bd-enhancing bacteria appeared to be relatively more abundant before the onset of the wet season across the three dendrobatoid species, this difference fell short from statistical significance (**Figure 5D**, *P* > 0.05), and we found no discernible difference in the proportion of Bd-enhancing bacteria among frog species (*P* > 0.05).

## Discussion

In this study, we assessed the role of two abiotic factors (i.e., soil pH and precipitation levels), seasonal, and host effects in shaping the amphibian skin microbiota diversity and predicted functional groups. To address our hypotheses, we sampled *D. auratus* across four sites differing in soil pH and precipitation levels, and three dendrobatoid frog species before and after the onset of the wet season within one site.

Our results demonstrate that variation in the skin microbiota of the Panamanian dendrobatoid frogs, *D. auratus, S. flotator*, and *A. talamancae*, from the Panama Canal region correlates with environmental variation in soil pH and changes in rainfall associated with the onset of the wet season. This is consistent with the hypothesis that these frogs depend on microbial environmental reservoirs to maintain their diverse skin microbiota (Fitzpatrick and Allison, 2014; Loudon et al., 2014; Kueneman et al., 2016a; Rebollar et al., 2016b). Thus, inhabiting an environment with conditions that promote higher microbial alpha diversity could translate into the frog’s skin being colonized by more microbial species. Typically, soil bacterial alpha diversity is expected to correlate with soil pH following a quadratic model, with highest diversity in nearly neutral soils with pH ≈ 6.0 than soils with pH <4.5 or >8 (Fierer and Jackson, 2006; Lauber et al., 2009; Zhalnina et al., 2015). However, our findings that *D. auratus* from the lowest soil pH site, Fort Sherman, had the highest alpha diversity and that frogs from Fort Sherman and Barro Colorado, which had the most extremely acidic and basic soil pHs, respectively, among our sample sites had the most differentiated bacterial communities, suggest that the frog host may nevertheless influence the composition of its skin microbiota. This finding is in line with evidence that amphibian skin microbiota can be composed disproportionately of microbes that are otherwise present in relatively low abundances in the environment (Walke et al., 2014; Rebollar et al., 2016b).

Although precipitation levels have no apparent effect on skin microbiota alpha diversity in *D. auratus*, they do appear to correlate with skin bacterial community dispersion patterns, as frogs sampled from sites with wetter climates showed lower between sample variation than frogs sampled from sites with drier climates. This is in line with the thought that moisture promotes more diverse environmental microbial communities (Lauber et al., 2009; Hartmann et al., 2014). The site differences may be explained both in terms of the relative abundances and the presence (or absence) of particular OTUs. Three of the bacterial classes that we found to be overrepresented, Actinobacteria, Gammaproteobacteria, and Betaproteobacteria, were previously reported to be more abundant in frogs from Bd-positive sites, whereas Sphingobacteria were abundant in frogs from a Bd-negative site (Rebollar et al., 2016b).

A dependence on microbial environmental reservoirs to maintain frog skin bacterial communities is further indicated by the significant change in skin bacterial alpha diversity, particularly in *S. flotator* at Pipeline Road, coincident with the onset of the wet season rains. The streamside habitat of these frogs tends to dry almost completely during the dry season but floods during the wet season, which may result in an increase in stress hormones, which has been related to a reduction in alpha diversity in the oral microbiota of certain species (Stothart et al., 2016). Our results also indicate that skin microbial communities of different species of frogs can become more similar with the onset of the wet season, which may be due to the more homogeneously damp environment after the start of the rains, allowing the frogs to forage more widely. Such seasonal changes in microbial community structure have also been reported in the skin microbiota of temperate ranid frogs (*Lithobates yavapaiensis*, Longo et al., 2015), and in the gut microbiota of other vertebrate groups (Keenan et al., 2013). Moreover, whether these seasonal patterns result in changes in the overall functional groups of the frog skin microbiota remained largely unexplored.

The redundancy that we found in microbial functional groups, whereby similar functions may be attributed to more than one bacterial OTU, may corroborate with evidence of an amphibian skin functional core (Hamady and Knight, 2009; Human Microbiome Project Consortium, 2012; Davis et al., 2017). From an evolutionary ecology perspective, such redundancy of microbial functional groups may be expected to be beneficial to the host in the face of potentially rapid microbial turnover (Loudon et al., 2014; Hughey et al., 2016), as an essential metabolic function, if carried out by only a single OTU could easily be lost, to the detriment of the host’s fitness. Such redundancy of ecologically relevant microbial functional groups has also been observed in soil (Wellington et al., 2003). In our study, the most abundant microbial functional groups were chloroplasts in Fort Sherman, aerobic chemoheterotrophs in Cerro Ancon, anoxygenic photoautotrophs in Pipeline Road, and human pathogens in Barro Colorado Island. The various bacteria representing these functional groups can exploit different carbon and energy sources (Balkwill et al., 1989; Johnston et al., 2009), which might indicate the position they occupy in the microbial biofilm and whether they interact predominantly with the frog host or with other microbes. The overabundance of human pathogens in frogs from Barro Colorado Island may also suggest that amphibians can play a role in the transmission of zoonotic diseases (Daszak et al., 2000) or, reciprocally, that human activities can affect the microbial communities of wildlife (Hacioglu and Tosunoglu, 2014). The shift in the most abundant functional groups from before to after the onset of the wet season from bacteria associated with photoautotrophy and nitrification to those associated to chemoheterotrophy and aerobic chemoheterotrophy suggests that when the environmental context of the host becomes more homogeneous, the skin microbiota follow a similar trend. That the microbial functional group differences we observe are ecologically relevant to the frog host remain, however, an assumption. How these functional groups actually interact with the host remains largely unknown.

The skin microbiota of these frogs may also be shaped by the properties of the frog’s skin, as well as the environment. All amphibians produce and sequester a rich variety of nitrogenous compounds, including alkaloids, in skin granular glands that could represent a strong microbial selective force. Indeed, alkaloids purified from anuran skin extracts have been found to act as antimicrobials in *in vitro* trials (Mina et al., 2015). Like other species of dart-poison frogs, *D. auratus* sequesters dietary-derived alkaloids in its skin to a far greater extent than either *S. flotator* or *A. talamancae*. The relative high abundances of aromatic compound degrading bacteria, such as *Rhodococcus, Nocardioides* and *Acinetobacter*, that we found in the skin microbiota of *D. auratus* compared to those of *S. flotator* and *A. talamancae* suggest that certain microbes might interact with the frog’s toxic alkaloids. This is similar to the case of the Midwife Toad, *Alytes obstetricans*, which secretes potent skin peptides that are also thought to play a role in shaping its microbiota (Davis et al., 2017). The potential for interactions between biogenically active amphibian skin compounds and amphibian skin microbiota regarding the frog’s toxicity and its immunity from disease may have considerable ecological and evolutionary implications.

Even though all the frogs we sampled tested negative for Bd, this chytrid amphibian pathogen is known to be present on both sides of the Panama Canal (Woodhams et al., 2008; Rebollar et al., 2014) and our results suggest that environmental factors may mediate the abundance of Bd-inhibitory and Bd-enhancing microbiota. Variation in the proportion of Bd-inhibitory bacteria in the skin of *D. auratus* correlated with mean soil pH, and decreasing proportions of Bd-enhancing bacteria in the skin of all three frog species in concert with the onset of the wet season, as we have found, may help explain seasonal variation observed in Bd prevalence (Lips et al., 2006; Longo et al., 2010; Whitfield et al., 2012). The lower proportions of Bd-inhibitory bacteria and higher proportions of Bd-enhancing bacteria we observed in the microbiota of *A. talamancae* compared to *D. auratus*, may signify the presence of differential effects on population persistence in these frogs (Lam et al., 2010). These findings may offer an explanation for previous suggestions that *A. talamancae* is more susceptible to Bd than *D. auratus* (Lips et al., 2006), and that susceptible frog species have distinct skin microbiota (Rebollar et al., 2016b).

Both the FAPROTAX database (Louca et al., 2016) and the Bd-interacting bacteria database (Woodhams et al., 2015) that we used to identify frog skin microbial functions have their limitations. As they are not exhaustive, they may be able to assign only a small percentage of discovered bacterial OTUs to one or more functional groups, enabling us to predict the function of less that 25% of the frog skin bacterial community. Furthermore, a particular bacterial OTU might carry out distinct functions depending on environmental context (Medina et al., 2017) and thus our predictions could be underestimating bacterial functional diversity. However, it is unlikely that we reported a bacterial functional group that was actually absent as most of these were largely redundant. Nevertheless, because both of these databases only consider cultured bacterial species with characterized functions, they may be considered superior to genomic approaches, like PICRUSt (Langille et al., 2013) or PAPRICA (Bowman and Ducklow, 2015), in minimizing Type I errors in predicting microbial functional groups.

We note that as both soil pH and precipitation levels are associated to the sampling sites, to be cautious we could interpret the differences discussed here as site differences. Similarly, given our reduced sample sizes and lack of seasonal replicates, we should be cautious when generalizing the results associated with the onset of the wet season. Despite these limitations, we present evidence that abiotic factors, site effects, and host effects shape bacterial skin community structure and predicted function in three tropical frog species.

The variability we observe in the skin microbiota of these three species of Panamanian frogs appears clearly to be correlated with abiotic factors, even though there may not yet be sufficient evidence to assign cause and effect. Frog skin microbial communities may plausibly be affected by interactions of constituent microbes with the environment, the host, and/or other co-occurring microbial species. Thus, there remains much more to be understood about such wildlife-associated microbiota and the complexities of their interactions with their amphibian hosts.

## Author Contributions

BV designed the study, collected the data, performed the analyses, and wrote the first version of the manuscript. DL, RI, and DG revised the manuscript. BV and DG prepared the figures. All authors read and approved the final manuscript.

## Conflict of Interest Statement

The authors declare that the research was conducted in the absence of any commercial or financial relationships that could be construed as a potential conflict of interest.
