# Supplementary material for: Environmental and Host Effects on Skin Bacterial Community Composition in Panamanian Frogs
Source: Front Microbiol. 2018 Feb 22;9:298. doi: 10.3389/fmicb.2018.00298 (PMC5826957; doi:10.3389/fmicb.2018.00298)
Supplement: Supplementary file 1 [file Table_1.docx]

**Supplementary Table S1. Full test statistics associated with the alpha, beta and functional diversity analyses.**

Statistically significant P-values are **bolded**, and values that might be biologically relevant (P<0.1) are *italicized*.

| **Dataset** | **Diversity measure** | **R model** | **Result** |
| --- | --- | --- | --- |
| ‘Dend.aura’ | Alpha  (Shannon) | ph.m1 <- lmer(Shannon ~ Soil.pH + Annual.Rainfall + Cumulative.15 + Life_stage + (1\|site_id)) | Type II Wald X^2^  Soil pH X^2^=3.9264  Df=1  **P=0.04753***  Annual.Rainfall  X^2^=0.3044  Df=1  P=0.58117  Cumulative.15  X^2^=0.6698  Df=1  P=0.41313  Life stage  X^2^=0.8792  Df=1  P=0.34843 |
|  | Alpha  (PD whole tree) | ph.m2 <- lmer(PD ~ Soil.pH + Annual.Rainfall + Cumulative.15 + Life_stage + (1\|site_id)) | Type II Wald X^2^  Soil pH X^2^=2.8811  Df=1  *P=0.08963*  Annual.Rainfall  X^2^=0.3334  Df=1  P=0.56369  Cumulative.15  X^2^=0.0770  Df=1  P=0.78136  Life stage  X^2^=0.7411  Df=1  P=0.38930 |
|  | Alpha  (Observed OTUs) | ph.m3 <- lmer(Observed ~ Soil.pH + Annual.Rainfall + Cumulative.15 + Life_stage + (1\|site_id)) | Type II Wald X^2^  Soil pH X^2^=1.2338  Df=1  P=0.2667  Annual.Rainfall  X^2^=0.1744  Df=1  P=0.6763  Cumulative.15  X^2^=0.0097  Df=1  P=0.9217  Life stage  X^2^=0.6240  Df=1  P=0.4296 |
|  | Beta  (Bray-Curtis) | pH.bray <- phyloseq::distance(pH1_rare, method="bray")  adonis(pH.bray ~ Soil.pH + Annual.Rainfall + Cumulative.15 + Life_stage , strata=site_id, permutations = 999) | Soil pH  F _1,50_= 2.4897  **P = 0.002****  Annual rainfall  F _1,50_= 3.9927  **P = 0.001*****  Cumulative 15  F _1,50_= 2.0581  **P = 0.026***  Life stage  F _1,50_= 1.1767  P = 0.247 |
|  | Beta  (UniFrac Weighted) | pH.uni.w <- phyloseq::distance(pH1_rare, method="unifrac", weighted=TRUE)  adonis(pH.uni.w ~ Soil.pH + Annual.Rainfall + Cumulative.15 + Life_stage , strata=site_id, permutations = 999) | Soil pH  F _1,50_= 2.3975  **P = 0.015***  Annual rainfall  F _1,50_= 5.1495  **P = 0.001*****  Cumulative 15  F _1,50_= 1.9552  *P = 0.058*  Life stage  F _1,50_= 0.4533  P = 0.947 |
|  | Beta  (UniFrac UnWeighted) | pH.uni.uw <- phyloseq::distance(pH1_rare, method="unifrac", weighted=FALSE)  adonis(pH.uni.uw ~ Soil.pH + Annual.Rainfall + Cumulative.15 + Life_stage , strata=site_id, permutations = 999) | Soil pH  F _1,50_= 1.25298  **P = 0.040***  Annual rainfall  F _1,50_= 1.22918  **P = 0.050***  Cumulative 15  F _1,50_= 1.32342  **P = 0.028***  Life stage  F _1,50_= 0.89125  P = 0.847 |
|  |  |  |  |
| ‘Pipeline’ | Alpha  (Shannon) | *A. talamancae*  t.test(Shannon~Wet.Season, var.equal=TRUE, alternative= "two.sided", paired=FALSE)  *D. auratus*  t.test(Shannon~Wet.Season, var.equal=TRUE, alternative= "two.sided", paired=FALSE)  *S. flotator*  t.test(Shannon~Wet.Season, var.equal=TRUE, alternative= "two.sided", paired=FALSE) | *A. talamancae*  t= 0.44  df= 4  P= 0.682  *D. auratus*  t= 0.28  df= 9  P= 0.785  *S. flotator*  t= 2.77  df= 4  **P=0.05*** |
|  | Alpha  (PD whole tree) | *A. talamancae*  t.test(PD~Wet.Season, var.equal=TRUE, alternative= "two.sided", paired=FALSE)  *D. auratus*  t.test(PD~Wet.Season, var.equal=TRUE, alternative= "two.sided", paired=FALSE)  *S. flotator*  t.test(PD~Wet.Season, var.equal=TRUE, alternative= "two.sided", paired=FALSE) | *A. talamancae*  t= 0.358  df= 4  P= 0.738  *D. auratus*  t= -0.486  df= 9  P=0.639  *S. flotator*  t= -1.639  df= 4  P= 0.177 |
|  | Alpha  (Observed) | *A. talamancae*  t.test(Observed~Wet.Season, var.equal=TRUE, alternative= "two.sided", paired=FALSE)  *D. auratus*  t.test(Observed~Wet.Season, var.equal=TRUE, alternative= "two.sided", paired=FALSE)  *S. flotator*  t.test(Observed~Wet.Season, var.equal=TRUE, alternative= "two.sided", paired=FALSE) | *A. talamancae*  t= 0.971  df= 4  P= 0.387  *D. auratus*  t= 0.031  df= 9  P=0.976  *S. flotator*  t= -0.804  df= 4  P= 0.466 |
|  | Beta  (Bray-Curtis) | season.bray <- phyloseq::distance(season_rare, method="bray")  adonis(season.bray ~ Wet.Season, strata=Species.Code, permutations = 999) | F _1,21_= 2.836  **P = 0.007**** |
|  | Beta  (UniFrac Weighted) | season.uni.w <- phyloseq::distance(season_rare, method="unifrac", weighted=TRUE)  adonis(season.uni.w ~ Wet.Season, strata=Species.Code, permutations = 999) | F _1,21_= 6.051  **P = 0.001***** |
|  | Beta  (UniFrac UnWeighted) | season.uni.uw <- phyloseq::distance(season_rare, method="unifrac", weighted=FALSE)  adonis(season.uni.uw ~ Wet.Season, strata=Species.Code, permutations = 999) | F _1,21_= 2.642  **P = 0.001***** |
| ‘Pipeline’ | Alpha  (Shannon) | sp.m1 <- lmer(Shannon ~ Species.Code + Wet.Season + (1\|Wet.Season)) | Type II Wald X^2^  Species.Code  X^2^=6.6345  Df=2  **P=0.03625***  Wet.Season  X^2^=1.1215  Df=1  P=0.28959 |
|  | Tukey’s | > summary(glht(sp.m1, linfct = mcp(Species.Code = "Tukey")), test = adjusted("holm")) | Dend.aura – Allo.tata P=0.125  Silv.flot – Allo.tala P=0.616  Silv.flot – Dend.aura **P=0.49*** |
|  | Alpha  (PD whole tree) | sp.m2 <- lmer(PD ~ Species.Code + Wet.Season + (1\|Wet.Season)) | Type II Wald X^2^  Species.Code  X^2^=3.7726  Df=2  P=0.1516  Wet.Season  X^2^=0.7238  Df=1  P=0.3949 |
|  | Alpha  (Observed OTUs) | sp.m3 <- lmer(Observed ~ Species.Code + Wet.Season + (1\|Wet.Season)) | Type II Wald X^2^  Species.Code  X^2^=1.8772  Df=2  P=0.3912  Wet.Season  X^2^=0.0266  Df=1  P=0.8704 |
|  | Beta  (Bray-Curtis) | sp.pip.bray <- phyloseq::distance(canal_rare1, method="bray")  adonis(sp.pip.bray ~ Species.Code , pipeline_data$Wet.Season, permutations = 999) | F _2, 22_ = 1.2267  P= 0.177 |
|  | Beta  (UniFrac Weighted) | Sp.pip.uni.w <- phyloseq::distance(canal_rare1, method="unifrac", weighted=TRUE)  adonis(season.uni.w ~ Species.Code, strata=season_data$Wet.Season, data=season_data, permutations = 999)  adonis(sp.pip.uni.w ~ Species.Code, strata=pipeline_data$Wet.Season, permutations = 999) | F _2, 22_ = 1.1059  P= 0.158 |
|  | Beta  (UniFrac UnWeighted) | sp.pip.uni.uw <- phyloseq::distance(canal_rare1, method="unifrac", weighted=FALSE)  adonis(sp.pip.uni.uw ~ Species.Code , strata= pipeline_data$Wet.Season, permutations = 999) | F _2, 76_ = 1.0616  P= 0.198 |
|  |  |  |  |
| ‘Dend.aura’ | Functional Diversity | On Bray-Curtis distances  adonis(bray.func.DAU ~ site_id, permutations = 999) | F_(3,53)_=6.132  **P<0.001***** |
| ‘Pipeline’ | Functional Diversity | On Bray-Curtis distances  adonis(bray.func.season ~ Wet.Season, strata=Species.Code, permutations = 999) | F_(1,23)_=4.387  **P<0.01**** |
|  |  |  |  |
| ‘Dend.aura’ | Bd-inhibitory proportions | glm(Bd.inh ~ site_id, family = quasibinomial) | X^2^ deviance residuals deviance=6.144  df=(57,3)  **P=0.002**** |
|  | Post-Hoc= multiple comparison of means |  | Tukey contrasts for linear hypotheses  Barro Colorado – Cerro Ancon P<0.05, Barro Colorado – Pipeline Road P<0.01, every other site comparison P>0.05 |
| ‘Dend.aura’ | Bd-enhancing proportions | glm(Bd.enh ~ site_id, family = quasipoisson) | X^2^ deviance residuals deviance=0.196, df=(57,3), **P=0.013*** |
|  | Post-Hoc= multiple comparison of means |  | Tukey contrasts for linear hypotheses  Fort Sherman – Cerro Ancon **P<0.05***, Fort Sherman – Barro Colorado **P<0.05***, every other site comparison *P*>0.05 |
| ‘Pipeline’ | Bd-inhibitory proportions | glm(Bd.inh ~ Wet.Season + Species.Code, family = quasibinomial) | Season  X^2^ deviance residuals=2.420  df=(22,1)  **P=0.035***  Frog Species  X^2^ residuals deviance=2.420  df=(20,2)  **P=0.011*** |
|  | Post-Hoc= multiple comparison of means |  | Tukey post-hoc analyses indicated that only *D. auratus* differ from *A. talamancae*  multiple comparisons of means: Tukey contrasts for linear hypotheses *D. auratus* – *A. talamancae* **P<0.01****; *D. auratus* – *S. flotator* **P<0.001*****; *S. flotator* – *A. talamancae* *P*>0.05 |
| ‘Pipeline’ | Bd-enhancing proportions | glm(Bd.enh ~ Wet.Season + Species.Code, family = quasipoisson) | Season  X^2^ residuals deviance=0.052  df=(22,1)  P>0.05  Frog Species  X^2^ deviance residuals=0.046  df=(20,2)  P>0.05 |
| ‘Pipeline’ | Bd-enhancing proportions | glm(Bd.enh ~ Wet.Season + Species.Code, family = quasipoisson) | Season  X^2^ residuals deviance=0.052  df=(22,1)  P>0.05  Frog Species  X^2^ deviance residuals=0.046  df=(20,2)  P>0.05 |
