# Supplementary figures and images for: Environmental and Host Effects on Skin Bacterial Community Composition in Panamanian Frogs
Source: Front Microbiol. 2018 Feb 22;9:298. doi: 10.3389/fmicb.2018.00298 (PMC5826957; doi:10.3389/fmicb.2018.00298)

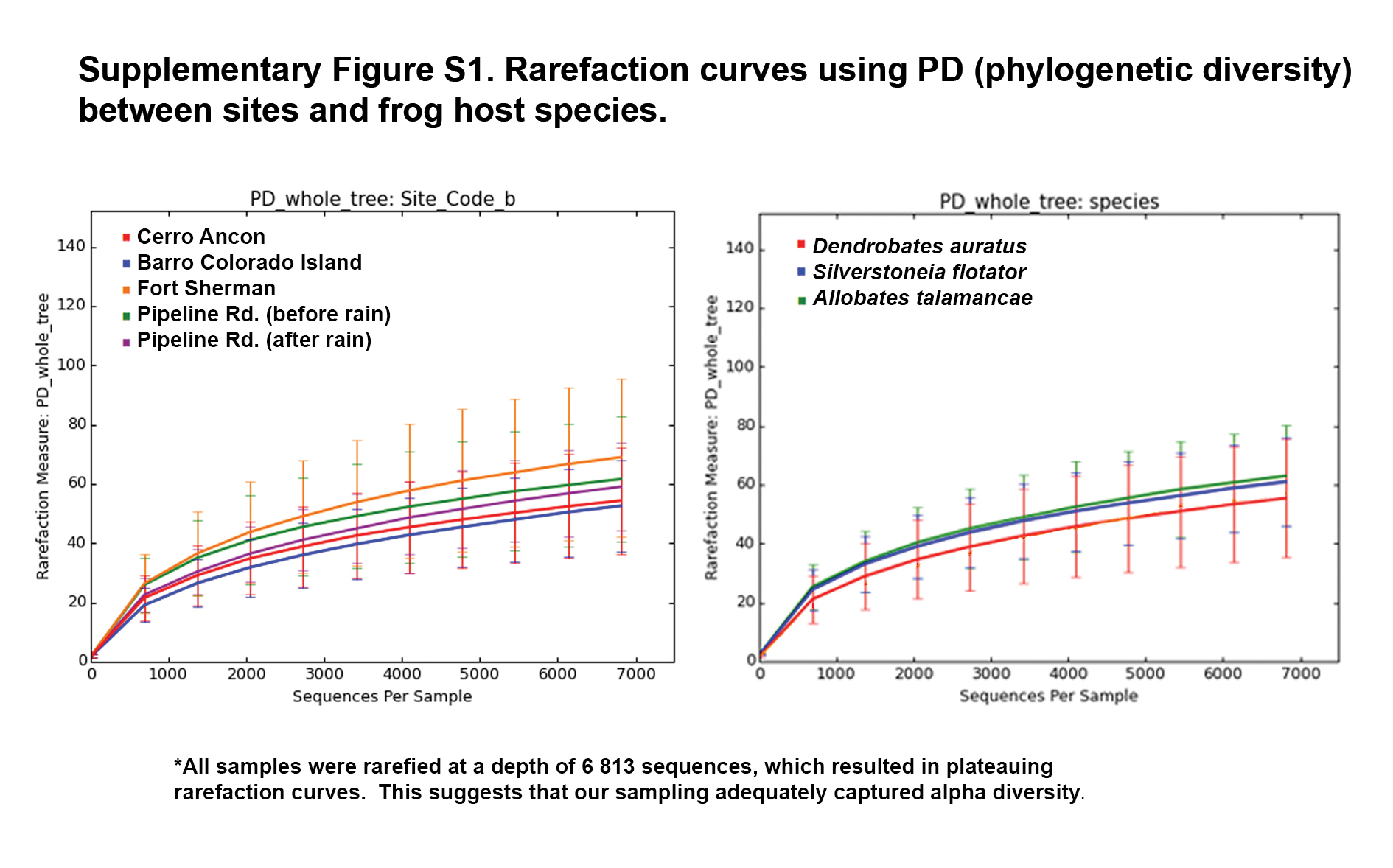

Supplement: Supplementary file 2 [file Image_1.JPEG]

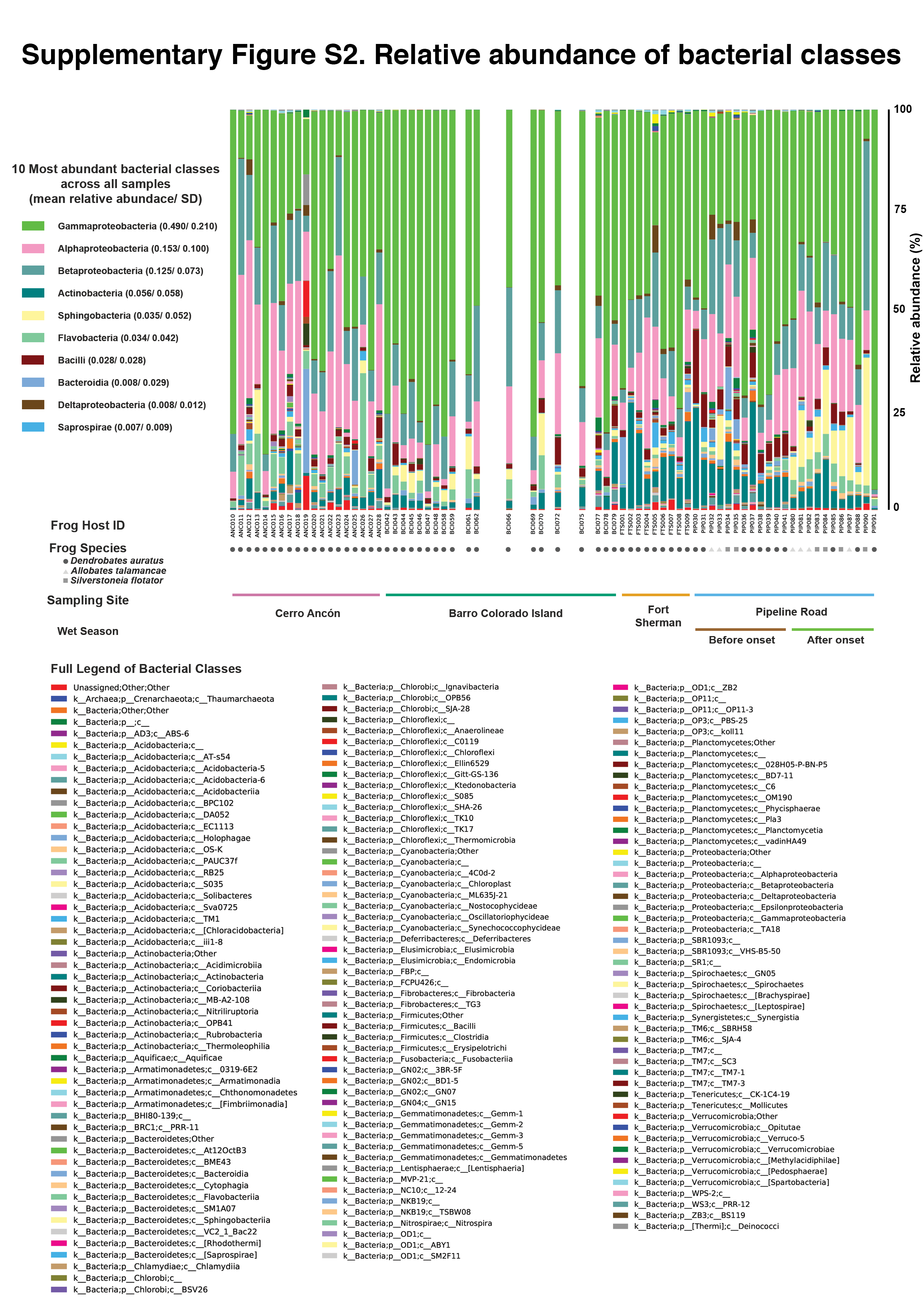

Supplement: Supplementary file 3 [file Image_2.png]
